# Prenatal Ultrasound Diagnosis of a Cyst of the Oral Cavity: An Unusual Case of Thyroglossal Duct Cyst Located on the Tongue Base

**DOI:** 10.1155/2016/7816306

**Published:** 2016-01-21

**Authors:** E. Rodríguez Tárrega, S. Fuster Rojas, R. Gómez Portero, S. Roig Boronat, G. Pérez Martínez, J. Zamora Prado, A. Perales Marín

**Affiliations:** Department of Obstetrics and Gynaecology, University Hospital La Fe, Avinguda de Fernando Abril Martorell, No. 106, 46026 Valencia, Spain

## Abstract

We describe a case of a lingual thyroglossal duct cyst diagnosed prenatally by ultrasound at 26 weeks of gestation. The follow-up ultrasound scans revealed no changes in the cyst measurement. Surgical treatment was performed without any complication 72 hours after delivery with good results.

## 1. Introduction

Thyroglossal duct anomalies are the most common malformation in the neck [1–7]; they can be found anywhere in the migration pathway of the thyroid gland from the floor of the mouth to its final location and consist in the persistence of embryological epithelial rests at this pathway.

Lingual presentation is uncommon. Published papers describe this type of presentation from 2.1 to 8% [3, 4, 7] and 70% of the cases correspond to a lingual thyroid and not to a persistent thyroglossal duct [8].

It is important to make a differential diagnosis with other anomalies. When prenatal diagnosis is made, a follow-up to detect cyst growth is recommended and its final size and characteristics will determine the mode of delivery.

## 2. Case Presentation

A 24-year-old primigravida with a singleton pregnancy was referred to our center because of the finding of an oral cyst diagnosed during a routine ultrasound at 26 weeks of gestation.

The medical history of the parents was unremarkable and they were nonconsanguineous.

The first and second trimester scans performed at 12 and 20 weeks of gestation in another center were normal.

Ultrasounds were performed using a GE Voluson 730 Expert.

The initial examination performed at 28 weeks of gestation showed a single male fetus with an oral cystic lesion of 18 × 10 mm, located under the tongue. The tumor appeared to be fluid and homogeneous, without solid components, with well-defined limits and it moved with the tongue movements. The most likely diagnosis with that ultrasound appearance was a congenital ranula or a thyroglossal duct cyst.

**Figure 1:**
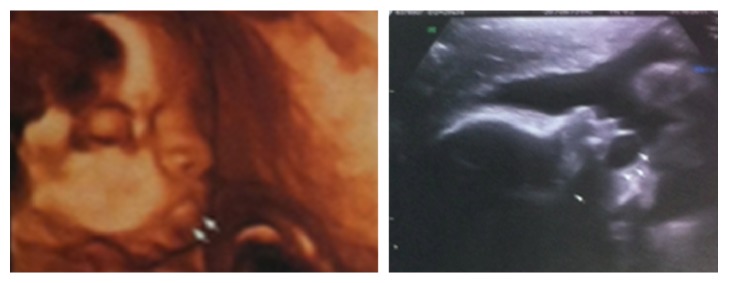
First examination shows an oral liquid cyst, assessed by 2D and 3D ultrasound.

The fetal anatomic study found no other abnormalities, except a left renal pelvic dilatation of 7.3 mm; fetal biometric parameters and amniotic fluid index were normal (Figure 1).

**Figure 2:**
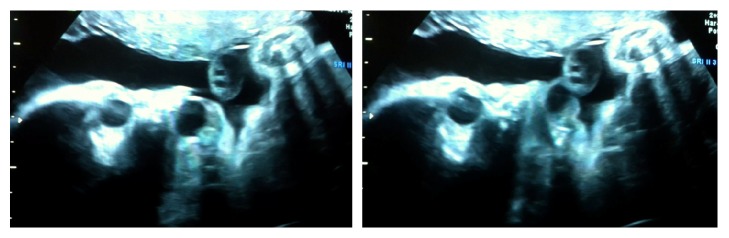
Ultrasound image showing how the tumor moves with the tongue movements and it is completely out of the fetal mouth.

Follow-up ultrasound scans performed every 2 weeks always showed appropriate fetal growth (75–90 percentile) and a normal amniotic fluid index. There were no changes in the measurements of the oral cyst and the most striking finding was that the fetus kept his mouth open during all examinations (Figure 2).

**Figure 3:**
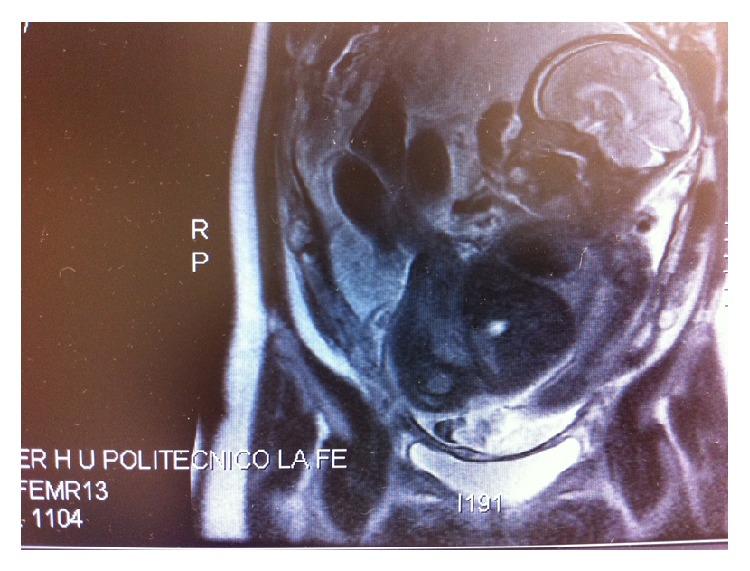
Magnetic resonance imaging of the fetal cyst.

Fetal magnetic resonance imaging was performed at 31 weeks and revealed a moderate left renal pelvic dilatation and an orofacial lesion of 18 × 14 mm located before the free edge of the tongue and above the lower lip, with cystic characteristics and the same echogenicity as the amniotic liquid, without evidence of fat or signal diffusion restriction. That image could correspond with a cyst probably dependent on the salivary glands (Figure 3).

At term, the oral tumor did not show any changes in measurements and characteristics, there was no evidence of polyhydramnios, and the stomach was visible; we also could see complete tongue movements and we occasionally observed that the fetus was able to close his mouth.

We discussed the mode of delivery in this case with a multidisciplinary team of pediatrics, anesthesiologists, and obstetricians and we decided to try a vaginal delivery in the first place, as the tumor did not seem to compromise the fetal airway.

Labor was induced at 40 weeks and a normal 4300 g male was born by caesarean section performed by failure to progress in labor. The Apgar score was 10/10 at 1 and 5 minutes of life.

**Figure 4:**
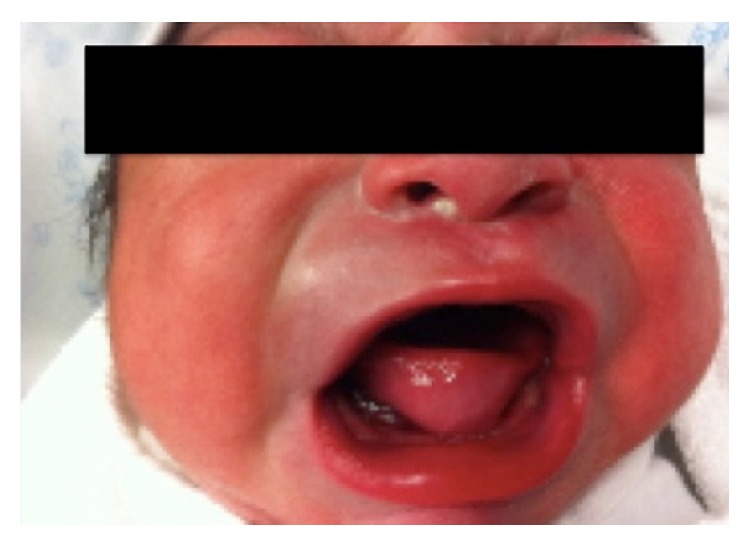
Male newborn with the tumor under the tongue.

The neonatal examination showed that the oral tumor was under the tongue with the same measurement seen at the intrauterine scans, without airway or digestive compromise, and the newborn did not require immediate admission to neonatal care unit (Figure 4).

72 hours after birth, the infant was admitted to the neonatal care unit due to a decrease of 600 g of weight and for excision of the cyst with a suspected diagnosis of congenital tongue mucocele. This procedure was performed without any complications and pathological definitive diagnosis was persistent thyroglossal duct of the tongue. Postoperative course was normal.

At the first medical examination when the infant was one month old, he had an adequate growth, uncomplicated breastfeeding and he was able to close his mouth completely.

The follow-up by endocrinologists at 4 and 8 months of life was normal. It included an ultrasound of the neck and thyroid gland and thyroid hormone determinations to rule out any aberrant thyroid tissue in other locations.

## 3. Discussion

Thyroglossal duct anomalies might not become evident until adult life [1] and they show a slight male preponderance and constitute 70% of all congenital cervical masses [2].

The thyroid gland appears in the third week of life as a well-defined endodermal sac between the first pair of pharyngeal pouches, attached to the pharynx by a narrower neck known as thyroglossal duct, which connects the primitive thyroid with the tongue temporarily. At six weeks this canal becomes solid and disappears. When this degeneration process does not occur, the partial or total persistence of the thyroglossal duct can result in fistulas or cyst formation [9].

The typical location for this type of cysts is the cervical midline. The lingual thyroglossal duct cysts are often located on the tongue base and its anterior presentation is poorly described in the literature [8, 10, 11]. They may be incidentally detected in child or adulthood [7, 12] or misdiagnosed as laryngomalacia disease by its pulmonary symptoms [1, 13].

Prenatal diagnosis of congenital head and neck masses is becoming more frequent with the improvement of ultrasound technology although it can be difficult to establish the etiology, which often is done by the histological exam. The magnetic resonance imaging can help us to know the precise location and extension of the tumor and so contribute to a better evaluation of the case.

Once the diagnosis is done, it should be necessary to monitor the cyst size, its mobility, and the amniotic fluid volume because a rapid tumor growth could hinder fetal swallowing and produce polyhydramnios, which increases the possibility of preterm delivery or sudden infant death by asphyxia if it is misdiagnosed [1, 11, 14]. We recommend monthly ultrasound assessment and if polyhydramnios or fast changes on the tumor are detected the obstetric surveillance must be closer, fortnightly or weekly.

The mode of delivery should be planned by a multidisciplinary team [4, 14] always keeping in mind the possible obstruction of the fetal airway that can be managed by an ex utero intrapartum treatment (EXIT) caesarean procedure. This involves establishing an airway before the fetomaternal circulation is interrupted. The small size of the cyst in our case, without changes throughout the gestation, allowed giving the option of a vaginal birth because we did not suspect the fetal airway to be blocked.

In the differential diagnosis, we must consider other formations such as congenital ranula, which was our suspected diagnosis, submandibular duct anomalies, and heterotopic gastric cysts [15]. In the case of cervical thyroglossal duct cyst, the simultaneous mobility with the thyroid gland can help in the differential diagnosis.

In the present case, the suspected pre- and postnatal diagnosis were a congenital ranula, because of the characteristics of the cyst, its mobility, and its location under the tongue and not on its base that is the usual location for the thyroglossal duct cyst.

The neonatal prognosis is usually good and uncomplicated and it depends on the tumor size, as shown in this case. An immediate excision after delivery could be required in cases of large cysts that block the newborn's airway or make the breastfeeding difficult.

A follow-up of these children by pediatrician and endocrinologists to test the thyroidal function is recommended.

## 4. Conclusions

The thyroglossal duct cyst of the tongue is an unusual finding. When a prenatal cyst in the oral cavity is diagnosed, an adequate follow-up is necessary to assess the size and growth potential of the cyst and possible associated complications. According to this, the mode of delivery will be decided, as well as the need for immediate action to ensure the airway of the newborn after delivery. Definitive diagnosis is achieved after histological study.
